# Robotic Versus Open Renal Transplantation in Obese Patients: Protocol for a Cost-Benefit Markov Model Analysis

**DOI:** 10.2196/resprot.8294

**Published:** 2018-03-08

**Authors:** Michele Molinari, Chethan Puttarajappa, Martin Wijkstrom, Armando Ganoza, Roberto Lopez, Amit Tevar

**Affiliations:** ^1^ Division of Transplant Surgery Department of Surgery University of Pittsburgh Medical Center Pittsburgh, PA United States; ^2^ University of Pittsburgh Transplant Centre University of Pittsburgh Medical Center Pittsburgh, PA United States; ^3^ Department of Medicine University of Pittsburgh Medical Center Pittsburgh, PA United States

**Keywords:** renal transplantation, obesity, cost benefit analysis, markov model

## Abstract

**Background:**

Recent studies have reported a significant decrease in wound problems and hospital stay in obese patients undergoing renal transplantation by robotic-assisted minimally invasive techniques with no difference in graft function.

**Objective:**

Due to the lack of cost-benefit studies on the use of robotic-assisted renal transplantation versus open surgical procedure, the primary aim of our study is to develop a Markov model to analyze the cost-benefit of robotic surgery versus open traditional surgery in obese patients in need of a renal transplant.

**Methods:**

Electronic searches will be conducted to identify studies comparing open renal transplantation versus robotic-assisted renal transplantation. Costs associated with the two surgical techniques will incorporate the expenses of the resources used for the operations. A decision analysis model will be developed to simulate a randomized controlled trial comparing three interventional arms: (1) continuation of renal replacement therapy for patients who are considered non-suitable candidates for renal transplantation due to obesity, (2) transplant recipients undergoing open transplant surgery, and (3) transplant patients undergoing robotic-assisted renal transplantation. TreeAge Pro 2017 R1 TreeAge Software, Williamstown, MA, USA) will be used to create a Markov model and microsimulation will be used to compare costs and benefits for the two competing surgical interventions.

**Results:**

The model will simulate a randomized controlled trial of adult obese patients affected by end-stage renal disease undergoing renal transplantation. The absorbing state of the model will be patients' death from any cause. By choosing death as the absorbing state, we will be able simulate the population of renal transplant recipients from the day of their randomization to transplant surgery or continuation on renal replacement therapy to their death and perform sensitivity analysis around patients' age at the time of randomization to determine if age is a critical variable for cost-benefit analysis or cost-effectiveness analysis comparing renal replacement therapy, robotic-assisted surgery or open renal transplant surgery. After running the model, one of the three competing strategies will result as the most cost-beneficial or cost-effective under common circumstances. To assess the robustness of the results of the model, a multivariable probabilistic sensitivity analysis will be performed by modifying the mean values and confidence intervals of key parameters with the main intent of assessing if the winning strategy is sensitive to rigorous and plausible variations of those values.

**Conclusions:**

After running the model, one of the three competing strategies will result as the most cost-beneficial or cost-effective under common circumstances. To assess the robustness of the results of the model, a multivariable probabilistic sensitivity analysis will be performed by modifying the mean values and confidence intervals of key parameters with the main intent of assessing if the winning strategy is sensitive to rigorous and plausible variations of those values.

## Introduction

Kidney transplantation (KT) is the best treatment strategy for patients with end-stage renal disease (ESRD). KT allows patients to return to a normal lifestyle with relatively few side effects from modern immunosuppression medications [1]. Several investigators have shown that from a societal point of view, KT is cost-effective [2-4]. However, these findings have been challenged by the increasing proportion of patients affected by obesity and renal failure and the introduction of costlier surgical technologies, such as robotic-assisted surgery. The constraints on health-care resources raise the question of the cost-benefit ratio of new medical or surgical therapies that are expensive or that provide a marginal benefit in comparison to already established therapies. Due to the lack of cost-benefit studies on the use of robotic-assisted renal transplantation versus open surgical procedure, the primary aim of our study is to develop a Markov model to analyze the cost-benefit of robotic surgery versus open traditional surgery for the treatment of obese patients undergoing renal transplantation. The secondary aim is to perform a cost-benefit analysis between the two competing surgical techniques.

### Innovation

The insufficient degree of freedom provided by non-articulating laparoscopic instruments and the two-dimensional view of conventional laparoscopic cameras have represented significant barriers preventing the widespread use of minimally invasive techniques for renal transplantation. However, most of those obstacles have been overcome by the introduction of robotic technologies such as the da Vinci surgical system (DVSS) (Intuitive Surgical, Mountain View, CA, USA). Robotic surgery allows intracorporal maneuvers that mirror the natural dexterity of surgeons’ hands with the additional advantages of eliminating the natural hand tremor [5-7]. Other significant benefits of using the robotic surgical system is the three-dimensional stereoscopic images and improved ergonomics for the primary surgeons in addition to the reduced discomfort for the patients who benefit from the minimally invasive approach and can return to their full functional capacity faster than open surgery [8].

### Limitations of Current Knowledge and Primary Aim of the Study

Due to the lack of cost-benefit studies on the use of robotic-assisted renal transplantation versus open surgical procedure, we aim to develop a mathematical model designed to analyze the cost-benefit of robotic surgery versus open surgery for the treatment of obese patients undergoing renal transplantation. Our primary aim is to assess the cost-benefit ratio for the health care payer’s perspective. The selection of obese recipients for this study is based on the current evidence indicating that, for this group of patients, robotic assisted renal transplantation is associated with a significant lower risk of wound complications, and therefore, lower costs for wound care and other expensive interventions such as repair of incisional hernias or use of open negative pressure wound dressings.

### Significance

The number of patients affected by renal insufficiency and obesity is growing, especially in North America where obesity has reached epidemic proportions [9]. Recent epidemiological data indicate that 20-50% of patients on dialysis are obese [10]. Obesity is associated with an increased risk of wound complications [9]. Wound infections are the most common nosocomial adverse events in patients who undergo complex surgical procedures or who are immunosuppressed or diabetic [11-13]. Most obese patients who undergo renal transplantation are diabetic and their risk of developing wound complications (infections, seromas, dehiscence, hernias) is increased further using immunosuppression medications that predispose to the development of infections and dehiscence or hernias. In obese recipients, wound complications have been estimated to range from 20-30% to 40% when body mass index (BMI) > 40 kg/m^2 [11-15]^.

### The Economic and Clinical Burden of Wound Infections

Wound infections, incisional hematomas, and seromas are predisposing factors for incisional hernias that, most of the times, will require surgical repair to prevent intestinal incarceration or strangulation, causing abdominal or back pain due to the disruption of balance between the anterior abdominal wall muscles and the paraspinal posterior musculature.

In theory, all wound complications are preventable. Yet, they still represent a significant clinical and economic burden to the health care system [16-20]. Wound infections are responsible for longer hospitalizations, increased costs for antibiotic therapy and topical wound care during the same admission and after discharge. In addition, patients who develop wound complications have decreased functional capacity and rely on the assistance of family members or other providers who need to take time off work to drive patients to their frequent clinic appointments [21]. More importantly, in obese renal transplant recipients, surgical site infections (SSIs) have been associated with lower graft survival [14].

### Wound Complications in Obese Patients

The higher incidence of wound complications in obese patients is multifactorial. Obese patients have a higher prevalence of diabetes that is a predisposing factor for delayed wound healing and to bacterial infections [22-25]. In addition, due to the extra-adipose tissue in the subcuticular space, obese patiens are prone to develop seromas that often become infected because of the suboptimal vascularization of the adipose tissue [26-29]. Furthermore, obese patients require longer incisions and their surgeries are, most of the time, longer with subsequent increased risk of tissue dissication and intraoperative contamination [30] that are predisposing factors for incisional hernias [31].

### Outcomes of Robotic Surgery in Renal Transplantation

During the period between June 2009 to December 2011, a prospective cohort of 39 obese patients underwent robotic kidney transplantation at the University of Illinois Hospital and Health Sciences System [30]. This cohort was compared to a similar group of patients who had open transplant surgery prior to June 2009. The two groups were matched for many clinical and sociodemographic characteristics [30]. Delayed graft function was observed in one patient (3.6%) who had robotic renal transplant compared to none in the open surgery group. Wound complications occurred in one patient (3.6%) who underwent robotic renal transplantation versus 8 (28.6%) who underwent open surgery (*P*=.004). There were no patient or graft losses within the first six months after transplantation and the two groups had comparable graft function with similar serum creatinine levels (1.5 mg/dL for robotic recipients versus 1.6 mg/dL for open surgery recipients) [30]. The authors analyzed possible differences in resource utilization between the two groups. Comparisons between robotic surgery and open surgery showed similar hospital stay (8.2 days versus 8.1 days respectively; *P*=.98), number of hospital days during the first 6 months after transplantation (14.3 days versus 15.8 days; *P*=.69), mean number of readmissions (1.6 versus 1.5; *P*=.82), percentage of reoperations during the first six months after surgery (0% versus 3.6%; *P*=.99), hospital costs for transplantation ($75,148 versus $60,552; *P*=.02) and total hospital costs over six months ($86,272 versus $66,487; *P*=.04) [30]. Oberholzer et al. suggested that the lower rate of SSIs observed with the minimally invasive approach was due to the fact that the classical suprainguinal incision located in a highly colonized skin area was replaced with a 7 cm periumbilical incision that was much smaller and located in a more favorable area of the abdominal wall. In their experience, only one patient with BMI of 54.5 kg/m^2^ and who underwent robotic assisted renal transplant developed an incisional hernia that required surgical repair.

### Costs and Benefits of Competing Therapies

In recent years, there has been a trend to move health services towards value-based organizations and to improve the cost-effectiveness of interventions by reducing costs and increasing the value of care [32-34]. Porter, one of the initiators of value-based care, defines value as the desired level of “health outcome achieved per dollar spent” [32]. By this definition, value-based care represents health services that create added value by optimizing how services are organized, delivered, and paid for in relation to the outcomes achieved [35]. The introduction of operative techniques that use more sophisticated and expensive equipment seems to work against the principle of cost-effective care and reduction of costs. However, this might not be the case if the initial higher costs are associated with better quality of life, shorter hospital stay, and reduced adverse outcomes. Wound infections are responsible for longer hospitalizations and decreased functional capacity of renal transplant recipients who require extramural nursing for the management of their wound-vacuum devices and local debridement and packing of their incisions. On the other hand, DVSS and other robotic surgical systems are associated with higher costs due to the initial acquisition of the primary robotic equipment in addition to the ongoing maintenance and buying of disposables that are needed for each surgery. Until the uncertainty on possible benefits of robotic surgery for patients undergoing renal transplantation is resolved by a randomized controlled trial, the development of a cost-benefit decision analysis models remain the best method to investigate whether robotic renal transplant surgery is cost-effective for patients at high risk of wound complications. We hypothesize that the increased intra-operative costs of using robotic surgery might be mitigated by the shorter hospital stay and decreased costs of wound care.

### Rationale for Cost-effectiveness and Cost-benefit Analysis

Cost-benefit analysis (CBA) in health care focuses on the analysis of the use of resources relative to expected medical benefits [36]. CBA plays an important role in selecting priorities or treatment strategies to be made in the presence of limited resources. CBA measures the correlation between costs and benefits using an equal unit of measure, usually monetary. It can be used to answer both technical and efficiency questions and can be applied to many sectors of the economy including health care. CBA and cost-effectiveness analysis (CEA) play an increasingly important role in the evaluation of interventions in modern health care systems [4]. With the advancement of treatment options available to treat common conditions, policy-makers and healthcare professionals are often required to choose among several competing therapies or surgical interventions that might be equally safe and effective but have different costs [37]. The purpose of CEA is comparison of alternative health interventions to make the most productive use of limited resources [36]. This has been made possible by modern computers able to handle multiple variables that populate probabilistic mathematical models.

## Methods

### Systematic Review of Clinical Effectiveness

With assistance of a librarian, electronic searches will be conducted to identify published studies on comparisons between open renal transplantation versus robotic assisted renal transplantation. Highly sensitive search strategies will include appropriate subject headings and text word terms, interventions under consideration, and specific study designs. No language restriction will be used but searches will be restricted from year 2000 onwards, reflecting the time of introduction of robotic assisted surgery. MEDLINE, MEDLINE In-Process & Other Non-Indexed Citations, EMBASE, BIOSIS, Science Citation Index, and Cochrane Central Register of Controlled Trials will be searched for primary studies, while the Cochrane Database of Systematic Reviews, the Database of Abstracts of Reviews of Effects, and the Health Technology Assessment database will be searched for reports of evidence syntheses. Reference lists of all included studies will be scanned to identify additional potentially relevant reports. Conference abstracts from meetings of the European, American, and British Urological Associations will be searched. Ongoing studies will be identified through searching Current Controlled Trials, ClinicalTrials.gov, the World Health Organization International Clinical Trials Registry and the National Institutes of Health Research Portfolio Online Reporting Tools Expenditures and Results. Websites of manufacturers, professional organizations, regulatory bodies, and the Health Technology Assessment will be checked to identify unpublished reports.

### Data Extraction Strategy

Two reviewers will independently screen titles and abstracts of all potentially relevant manuscripts. Full-text copies will be obtained whenever possible. Necessary variables found in the literature will be used to populate the mathematical model. Central tendency values and their variances will be used to create distributions used in the model. Probabilistic sensitivity analysis will be performed to assess the critical variables that influence the results of the model. For variables for which values are still unknown, we will elicit expert opinions to create plausible distributions. Alternatively, we will extract values reported in scientific publications that did not include transplant recipients but that used comparable surgical techniques or interventions. For example, variables associated with the costs of using robots in renal transplantation are unavailable. However, there are several observational studies and systematic reviews that analyzed the costs of robotic-assisted prostatectomies or partial nephrectomies that can be used for our model [38,39]. Similarly, costs of treatment of surgical site infections [19], repair of incisional hernias [40], and utility of patients undergoing incisional hernia repair [20] will be extracted from non-transplant scientific literature as we will assume that these values are applicable to our study population.

### Identification of Costs and Benefits

In this part of the study, transplant surgeons, robotic surgeons, and transplant nephrologists will create a list all the possible costs that might be associated with open and robotic assisted renal transplant surgery. After reaching a state of saturation where no further costs are identified, investigators and a representative sample of individuals who require renal replacement therapy will list all the potential benefits for the two competing surgical interventions. Costs and benefits of the two interventions will be captured for the perioperative period. Since the costs of immunosuppression medications and follow-up appointments occurring after renal transplantation are similar for both groups of patients, these costs will not be included in our final analysis. On the other hand, due to the expected differences in the incidence of wound complications leading to incisional hernias between robotic versus open renal transplantation, the added costs for the care of the repair of incisional hernias will be included and added to the operative costs of transplant surgery. We will assume that the costs for the care of wound complications and repair of incisional hernias between the two groups of patients will be the equivalent.

### Assignment of Monetary Value to the Costs

Costs associated with the two surgical techniques will incorporate the expenses of the resources used for the operations (eg, operative equipment, operative room time, and disposables). Training cost for surgeons will not be included as it depends on many variables including the overall level of experience of the surgeons, the number of hours spent for training on the robotic platform by the surgeons, and the maintenance costs for the training robotic system. Also, we will not include the costs to train operative nurses and technicians, anesthesia, and other health care providers working in the operating room to reach adequate proficiency in robotic assisted surgery. Similarly, because the preoperative workup is comparable for both groups, these costs will not be considered in our analysis.

### Assignment of Monetary Value to the Benefits

Monetary values associated to the benefits of each surgical technique will be obtained from studies already published in peer-reviewed scientific journals. We will include the costs of obese patients who might remain on renal replacement therapy since some transplant programs will not consider them candidates for renal transplantation unless their BMI is lower than 40. To obtain pertinent costs, a systematic search of the scientific literature will be performed with the assistance of one of the librarians at the University of Pittsburgh or University of Pittsburgh Medical Center. When unavailable, monetary values will be obtained using unpublished data from the University of Pittsburgh Medical Center or from suitable hospital accounting services. Additionally, there will be intangible, or soft, benefits associated to overall patients’ satisfaction, different levels and duration of perioperative discomfort, cosmetic results, time to full recovery, potential publicity, and marketing value associated with robotic surgery for either the hospital or the surgical team. To address how these intangible benefits should be measured and whether they should be included in the computerized model, all the members of our research team and a representative sample of patients requiring renal replacement therapy will be invited to a Delphi session to stimulate ideas and solutions based on sound clinical and methodological decisions. The Delphi Technique is a method used to estimate the likelihood and outcome of future events or to estimate probabilities or values that are unknown or not measurable [41,42]. A group of experts exchange views, and each independently gives estimates and assumptions to a facilitator who reviews the data and issues a summary report. The group members discuss and review the summary report and give updated forecasts to the facilitator, who again reviews the material and issues a second report. This process continues until all participants reach a consensus. In case consensus among the members of the research team is not reached, we will consult with other stakeholders and experts in decision analysis within the school of medicine at the University of Pittsburgh.

### Creation of Decision Analysis Tree

A decision analysis model will be developed to simulate a randomized controlled trial comparing three interventional arms: A) continuation of renal replacement therapy for patients who are considered non-suitable candidates for renal transplantation due to obesity; B) transplant recipients undergoing open transplant surgery; and C) transplant patients undergoing robotic-assisted renal transplantation. TreeAge Pro 2017 R1 (TreeAge Software, Williamstown, MA, USA) will be used to create a Markov model and microsimulation will be used to compare costs and benefits for the two competing surgical interventions (Multimedia Appendix 1).

### Patient Population, Model, and Variables

The model will simulate a randomized controlled trial of adult (age ≥ 18) obese patients affected by end-stage renal disease undergoing renal transplantation. The absorbing state (final state) of the model will be patients’ death from any cause. By choosing death as the absorbing state, we will be able simulate the population of renal transplant recipients from the day of their randomization to transplant surgery or continuation on renal replacement therapy to their death and perform sensitivity analysis around patients’ age at the time of randomization to determine if age is a critical variable for CBA or CEA comparing renal replacement therapy, robotic-assisted surgery, or open renal transplant surgery.

Obesity will be defined as patients’ BMI higher than 30 according to the World Health Organization classification [43,44]. For simplicity, the model will not simulate the possibility of patients assigned to the robotic surgery to cross arm and be converted to open surgery. Variables of the model will include: costs for robotic and open surgery, costs of remaining on renal replacement therapy, cost associated with the development of wound infections and hernia repair, probabilities of developing surgical site infections for both groups, probabilities of developing incisional hernias after uncomplicated robotic and uncomplicated open renal transplantation, and probability of developing incisional hernias after developing surgical site infections after robotic and open renal transplantation. A summary of some of the variables and ranges that will be used in the model are reported in Multimedia Appendix 2. The model will simulate the entire life span of patients included in the study until their death. For simplicity, the probability of developing incisional hernia requiring surgical repair after renal transplantation will be limited to two events only. Expected survival of each patient will be estimated from survival tables of individuals living in North America adjusted for their age at the time of inclusion.

### Sensitivity Analysis

After running the model, one of the three competing strategies will result as the most cost-beneficial or cost-effective under common circumstances. To assess the robustness of the results of the model, a multivariable probabilistic sensitivity analysis will be performed by modifying the mean values and confidence intervals of key parameters with the main intent of assessing if the winning strategy is sensitive to rigorous and plausible variations of those values. The rationale of sensitivity analysis in decision analysis is summarized in Textbox 1.

**Uses and contribution of sensitivity analysis for cost-benefit and cost-effectiveness analysis.**

Primary aims

- Testing the robustness of an optimal solution.
- Identifying critical values, thresholds or break-even values where the optimal strategy changes.
- Identifying sensitive or important variables.
- Investigating sub-optimal solutions.
- Developing flexible recommendations which depend on circumstances.
- Comparing the values of simple and complex decision strategies.
- Assessing the “riskiness” of a strategy or scenario.

Communication

- Making recommendations more credible, understandable, compelling or persuasive.
- Allowing decision makers to select assumptions.
- Conveying lack of commitment to any single strategy.

Increased Understanding or Quantification of the System

- Estimating relationships between input and output variables.
- Understanding relationships between input and output variables.
- Developing hypotheses for testing

Model Development

- Testing the model for validity or accuracy.
- Searching for errors in the model.
- Simplifying the model.
- Calibrating the model.
- Coping with poor or missing data.
- Prioritizing acquisition of information

## Results

The model will simulate a randomized controlled trial of adult obese patients affected by end-stage renal disease undergoing renal transplantation. The absorbing state of the model will be patients’ death from any cause. By choosing death as the absorbing state, we will be able simulate the population of renal transplant recipients from the day of their randomization to transplant surgery or continuation on renal replacement therapy to their death, and perform sensitivity analysis around patients’ age at the time of randomization to determine if age is a critical variable for CBA or CEA comparing renal replacement therapy, robotic-assisted surgery, or open renal transplant surgery. After running the model, one of the three competing strategies will result as the most cost-beneficial or cost-effective under common circumstances. In the discussion, we will summarize the results of our study and put them in the contest of the current knowledge on the value of robotic-assisted minimally invasive renal transplantation. We expect that, for some groups of patients, robotic surgery will be the most cost-effective treatment. The strength and limitations of our study will be presented and we will assess if our study could lead to the development of future research projects.

## Discussion

After running the model, one of the three competing strategies will result as the most cost-beneficial or cost-effective under common circumstances. To assess the robustness of the results of the model, a multivariable probabilistic sensitivity analysis will be performed by modifying the mean values and confidence intervals of key parameters with the main intent of assessing if the winning strategy is sensitive to rigorous and plausible variations of those values.

### Data Sharing

Sharing of data generated by our study is an essential part of our proposal. We would wish to make our results available to the community of scientists interested in robotic surgery, minimally invasive surgery, and transplantation to avoid unintentional duplication of research. We would welcome collaboration with other researchers within the University of Pittsburgh and from other institutions interested in CBA and CEA of new technologies in surgery. From this project, we expect that approximately two presentations will be delivered at national or international meetings. In addition, it is our explicit intention that the results of our study will be made readily accessible to the scientific community after the final analysis of the data generated by our mathematical model through publications in peer-reviewed journals.

## Appendix

Multimedia Appendix 1 Graphical representation of the decision analysis tree that will be used to perform a cost-benefit analysis between remaining on renal replacement therapy, robotic-assisted minimally invasive renal transplantation, and open surgery renal transplantation for obese patients affected by end-stage renal disease.

Multimedia Appendix 2 Summary of all variables that will be used in the mathematical model to perform a cost-benefit analysis between robotic-assisted minimally-invasive renal transplantation versus open surgery. All the variables were extracted from the most recent scientific literature.

## Abbreviations

BMI: body mass index
CBA: cost-benefit analysis
CEA: cost-effectiveness analysis
DVSS: da Vinci surgical system
ESRD: end-stage renal disease
KT: kidney transplantation
SSI: surgical site infection
