# Supplementary figures and images for: Robotic Versus Open Renal Transplantation in Obese Patients: Protocol for a Cost-Benefit Markov Model Analysis
Source: JMIR Res Protoc. 2018 Mar 8;7(3):e74. doi: 10.2196/resprot.8294 (PMC5865002; doi:10.2196/resprot.8294)

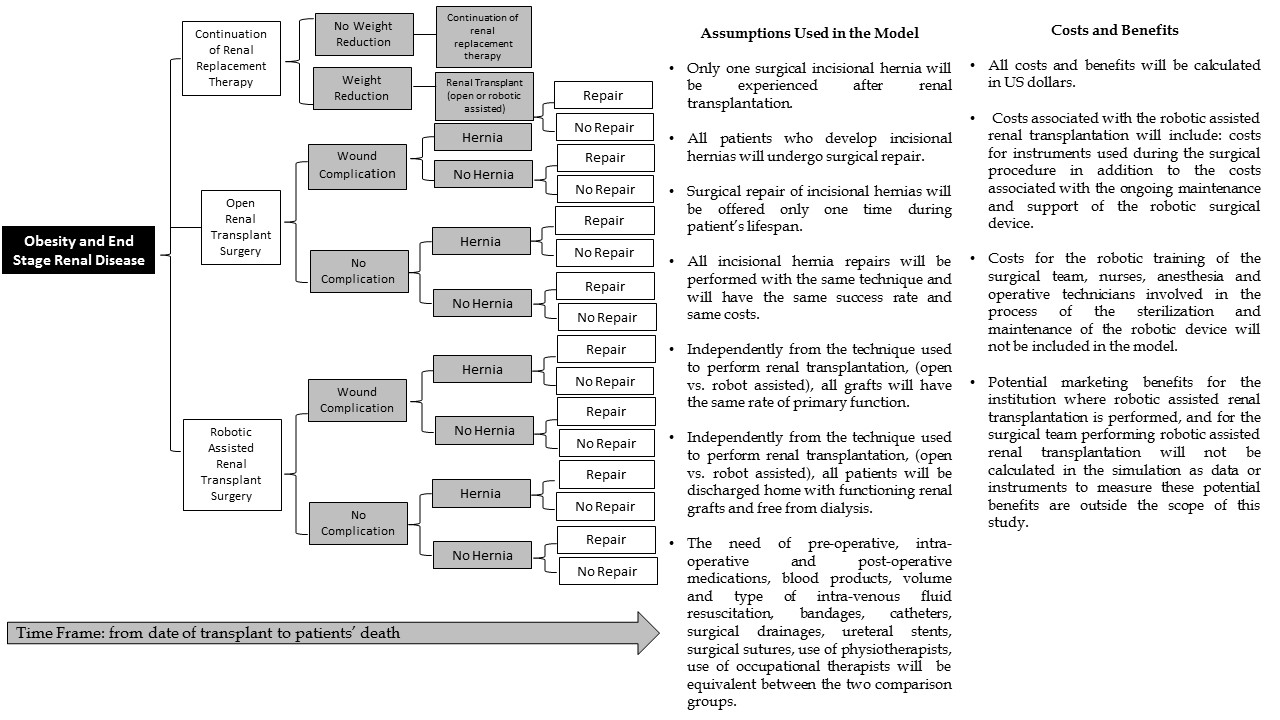

Supplement: Multimedia Appendix 1 [file resprot_v7i3e74_app1.png]
