# Supplementary material for: Robotic Versus Open Renal Transplantation in Obese Patients: Protocol for a Cost-Benefit Markov Model Analysis
Source: JMIR Res Protoc. 2018 Mar 8;7(3):e74. doi: 10.2196/resprot.8294 (PMC5865002; doi:10.2196/resprot.8294)
Supplement: Multimedia Appendix 2 [file resprot_v7i3e74_app2.pdf]

Table 1. Summary of all variables that will be used in the mathematical model to perform a cost-benefit analysis between robotic assisted minimally invasive renal transplantation versus open surgery. All the variables were extracted from the most recent scientific literature.

Authors	Journal, Year	Title	Surgery	Costs or Charges	Robotic	Laparoscopic	Open
Seideman et al. [45]	Journal of Endourology, 2012	Cost comparison of Robot-Assisted and Laparoscopic Pyeloplasty	Pyeloplasty	Purchase price for da Vinci robot	$1,500,000	$0	-
				Annual maintenance	$150,000	$0	-
				Robot cost per case	$1,214	$0	-
				Operating room costs	$2,990	$2,772	-
				Operating room costs per minute	$12.90	$12.90	-
				Equipment cost	$406	$1,375	-
				Recovery room	$270	$270	-
				Direct cost	$9,065	$10,635	-
Ferguson et al. [39]	Journal of Endourology, 2012	Cost Analysis of Robot-Assisted Laparoscopic Versus Hand-Assiste Laparoscopic Partial Nephrectomy	Partial Nephrectomy	Postoperative hospital costs (Range $3683-$5058)	$4,371	$5,984	$6,804
				Surgeon fees	$1,719	$1,719	$1,719
				Operating room costs	$7,276	$5,708	$5,017
				Operating room times (min.)	243	236	250
				Disposables	$669	$1,009	$0
				Overall costs	$13,439 (12,618-14,260)	$13,560 (12,149-14,971)	-
				Length of stay (days)	2.45 (2.07-2.83)	3.48 (2.99-3.99)	3.96 (3.35-4.5)
Ahmed et al.[38]	BJUI International, 2012	Assessing the Cost Effectiveness of Robotics in Urological Surgery - A Systematic Review	Prostatic surgery	Lotan et al. 2004 (total cost)	$7,280	$6,041	$5,554
				Lotan et al. 2005 (professional cost)	$1,688	$1,688	$1,594
				Lotan et al. 2006 (surgical equipment)	$533	$1,705	$75
				Lotan et al. 2007 (operating room)	$2,204	$2,876	$2,428
				Scales et al. 2005 (total cost)	$8,929	-	$8,146
				Scales et al. 2005 (professional cost)	$2,173	-	$1,787
				Scales et al. 2005 (surgical equipment)	$1,704	-	$575
				Scales et al. 2005 (operating room)	$2,183	-	$8,929
				Burgess et al. 2006	$39,315	$29,771	$31,518
				Burgess et al. 2006 (operative charges)	$25,443	$16,320	$16,522
				Burgess et al. 2006 (nonoperative charges)	$13,782	$13,451	$14,663
				Mouraviev et al. 2007 (total cost)	$10,047	$10,536	$10,704
				Mouraviev et al. 2007 (surgical equipment)	$3,441	$2,788	$2,471
				Lotan et al. 2010 (total cost)	$10,269	$8,557	$6,473
				Lotan et al. 2010 (operative charges)	$5,670	$4,021	$6,988
				Lotan et al. 2010 (non-operative charges)	$2,887	$2,452	$3,281

				Lotan et al. 2010 (professional charges)	$2,662	$2,250	$3,007
				Joseph et al. 2008 (total cost)	$5,410	$3,876	$1,870
				Joseph et al. 2008 (professional costs)	$494	$832	$330
				Joseph et al. 2008 (surgical equipment)	$4,805	$2,933	$1,429
				Bolenz et al. 2010 (total costs)	$6,752	$5,687	$4,437
				Bolenz et al. 2010 (surgical equipment)	$2,015	$725	$185
				Bolenz et al. 2010 (operating room costs)	$2,798	$2,453	$1,611
			cystectomy	Smith et al. 2010 (total costs)	$16,248	-	$14,608
				Lee et al. 2009 (total costs)	$22,685	-	$20,719
				Lee et al. 2009 (total costs)	$20,862	-	$19,057
				Lee et al. 2009 (total costs)	$20,659	-	$20,659
				Lee et al. 2009 (total costs)	$22,102	-	$22,697
				Lee et al. 2009 (operative charges)	$19,034	-	$18,303
				Lee et al. 2009 (operative charges)	$20,190	-	$20,178
				Lee et al. 2009 (operative charges)	$20,862	-	$19,057
				Lee et al. 2009 (professional)	$2,271	-	$2,271
				Lee et al. 2009 (professional)	$2,442	-	$2,442
Chatterjee et al. [40]	Annals of Plastic Surgery, 2015	Complex Ventral Hernia Repair Using Component Separation With or Without Biologic Mesh	Incisional Hernia Repair	Successful incisional hernia repair with biologic mesh			$7,983
				Successful incisional hernia repair without biologic mesh			$7,715
				Wound infection without biologic mesh			$10,471
				Wound infection with biologic mesh			$10,740
				Wound dehiscence without biologic mesh			$11,103
				Wound dehiscence with biologic mesh			$11,372
				Hernia recurrence without biologic mesh			$14,352
				Hernia recurrence with biologic mesh			$14,620
Fisher et al. [46]	Plastic and Reconstructive Surgery, 2016	A Cost-Utility Assessment of Mesh Selection in Clean-Contaminated Ventral Hernia Repair	Incisional Hernia Repair	Successful repair of incisional hernia with synthetic mesh			$11,226 (range $9,542-$12,910)
				Successful repair of incisional hernia with biologic mesh			$17,958 (range $15,264-$20,651)
				Recurrent hernia with successful repair with synthetic mesh			$22,183 (range $18,856-$25,511)

				Recurrent hernia with successful repair with biologic mesh	$28,915 (range $24,578-$33,252)
				Superficial wound infection (cellulitis) with synthetic mesh	$11,266 (range $9,576-$12,956)
				Superficial wound infection (cellulitis) with biologic mesh	$17,998 (range $15,298-$20,698)
				Wound infection requiring IV antibiotics (with synthetic mesh)	$17,405 (range $14,794-$20,016)
				Wound infection requiring IV antibiotics (with biologic mesh)	$24,137 (range $20,516-$27,757)
				Wound infection requiring reoperation (with synthetic mesh)	$23,772 (range $20,206-$27,337)
				Wound infection requiring reoperation (with biologic mesh)	$30,504 (range $25928-$35,079)
				Recurrence rate of incisional hernia after repair with synthetic mesh	17% (range 15-20)
				Recurrence rate of incisional hernia after repair with biologic mesh	14% (range 12-16)
				Reoperation after recurrence with synthetic mesh	88% (range 74-100)
				Reoperation after recurrence with biologic mesh	76% (range 64-87)
Chopra et al.[21]	Plastic and Reconstructive Surgery, 2016	The Economic Impact of Closed-Incision Negative-Pressure Thereapy in High-Risk Abdominal Incisions: A Cost-Utility Analysis	Ventral Hernia Repair	Cost of successful incisional hernia repair	$784
				Cost of surgical site infection	$21,569
				Hospital stay for successful incisional hernia repair	2.3 days
				Hospital stay for successful incisional hernia complicated by surgical site infection	9.7 days
				Average home therapy for surgical site infection	28 days
				Cost for standard dressings for wound care	$5,170
				Cost for closed-incision negative pressure therapy	$3,004
				Utility for uncomplicated incisional hernia repair	0.7
				Utility for complicated incisional hernia repair	0.49
Zimlichman et al. [47]	JAMA Intern Med, 2013	Health Care-Associated Infections: A Meta-Analysis of Costs and Financial Impact on the US Health Care System	Abdominal Surgery	Average attributable cost per surgical-site infection in 2012 in the USA	$20,785
Fisher et al. [31]	Annals of Surgery, 2016	A Risk Model and Cost Analysis of Incisional Hernia After Elective Abdominal Surgery Based on 12,373 Cases	Abdominal Surgery	Incidence of surgically treated incisional hernias	3.50%
				Incidence of superficial infection	2.3-4.3%
				Incidence of wound dehiscence	0.4-0.7%
				Incidence of seroma	0.2-0.3%
				Incidence of hematoma	0.7-1.2%
				Incidence of hernia after superficial infection	7.6-9.3%

				Incidence of hernia after wound dehiscence	6.40%
				Incidence of hernia after seroma	11.50%
				Incidence of hernia after hematoma	1.30%
				Total cost for incisional hernia repair (SD)	$27,065 (51,805)
				Total readmission cost (SD)	$34,489 (60,901)
				Risk of recurrent hernia after repair	10-20%
				Cost for readmission after hernia repair	$57,267 (78,929)
				Cost for 2 hernia repair	$98,414 (119,765)
